# IMPACT OF BODY POSITION AND ARM USE ON EYE DROP INSTILLATION IN OLDER ADULTS

**DOI:** 10.1093/geroni/igad104.2931

**Published:** 2023-12-21

**Authors:** Tanner Trenary, Andrew Droste, Rachel Logue Cook, Stephen Cain, Alanson Sample, David Burke, Paula Anne Newman-Casey, Susan Brown

**Affiliations:** University of Michigan, Ann Arbor, Michigan, United States; Michigan Medicine, Ann Arbor, Michigan, United States; University of Michigan, Ann Arbor, Michigan, United States; West Virginia University, Morgantown, West Virginia, United States; University of Michigan, Ann Arbor, Michigan, United States; Michigan Medicine, Ann Arbor, Michigan, United States; Michigan Medicine, Ann Arbor, Michigan, United States; University of Michigan, Ann Arbor, Michigan, United States

## Abstract

Visual impairments in older adults are associated with declines in physical, cognitive and social functioning, often leading to a loss of independence and quality of life. Increased age is a major risk factor, with conditions such as glaucoma having a 7-fold increase in prevalence from the 50s to the 80s (Klein & Klein, 2013). Treatment in the form of eye drops is commonly prescribed yet suboptimal adherence can significantly impact outcomes (Newman-Casey et al., 2015). The purpose of this preliminary study was to determine the effects of body posture and hand use on eye drop instillation success in a sample of 20 healthy, non-eye drop users (mean age: 70.0 +/- 3.7 y). Participants administered eyes drops without instruction while seated, standing and in a supine position. Video analysis was used to determine the number of attempts before successful instillation occurred (failed attempts) as well as whether participants used one or two hands. Despite published recommendations to use both hands (Davis et al, 2018), only 65% adopted a bimanual approach. The percentage of failed attempts was lowest in the supine (11.6%) compared to the seated (25.7%) or standing (21.2%) positions. Pinch strength was not correlated with successful instillation as has been previously reported (Naito et al., 2021), possibly due to participants’ overall level of physical functioning. Based on these results, a supine position is recommended for eye drop instillation where the head and neck are stabilized.

